# Supplementary material for: Role and mechanism of NCAPD3 in promoting malignant behaviors in gastric cancer
Source: Front Pharmacol. 2024 Apr 22;15:1341039. doi: 10.3389/fphar.2024.1341039 (PMC11070777; doi:10.3389/fphar.2024.1341039)
Supplement: Supplementary file 11 [file DataSheet2.ZIP › GSEA/immunologic/my_analysis.Gsea.1599462003817/GSE24142_ADULT_VS_FETAL_DN2_THYMOCYTE_UP.html]

Details for gene set GSE24142\_ADULT\_VS\_FETAL\_DN2\_THYMOCYTE\_UP[GSEA]

|  || Dataset | filtered\_dataset.sample\_info.cls#WT\_versus\_NCAPD3\_MUT |
| Phenotype | sample\_info.cls#WT\_versus\_NCAPD3\_MUT |
| Upregulated in class | NCAPD3\_MUT |
| GeneSet | GSE24142\_ADULT\_VS\_FETAL\_DN2\_THYMOCYTE\_UP |
| Enrichment Score (ES) | -0.58107823 |
| Normalized Enrichment Score (NES) | -2.5558462 |
| Nominal p-value | 0.0 |
| FDR q-value | 0.010544718 |
| FWER p-Value | 0.064 |
Table: GSEA Results Summary

  

Fig 1: Enrichment plot: GSE24142\_ADULT\_VS\_FETAL\_DN2\_THYMOCYTE\_UP      
 Profile of the Running ES Score & Positions of GeneSet Members on the Rank Ordered List

  

| SYMBOL | TITLE | RANK IN GENE LIST | RANK METRIC SCORE | RUNNING ES | CORE ENRICHMENT || 1 | 3433 | IFIT2 | 608 | 0.418 | -0.3851 | No |
| 2 | 3106 | HLA-B | 855 | -0.258 | -0.5301 | No |
| 3 | 23608 | MKRN1 | 927 | -0.352 | -0.5379 | Yes |
| 4 | 25901 | CCDC28A | 946 | -0.366 | -0.5058 | Yes |
| 5 | 54498 | SMOX | 965 | -0.384 | -0.4716 | Yes |
| 6 | 687 | KLF9 | 996 | -0.402 | -0.4438 | Yes |
| 7 | 901 | CCNG2 | 998 | -0.405 | -0.3948 | Yes |
| 8 | 51646 | YPEL5 | 1014 | -0.413 | -0.3549 | Yes |
| 9 | 3914 | LAMB3 | 1015 | -0.414 | -0.3041 | Yes |
| 10 | 1628 | DBP | 1029 | -0.429 | -0.2608 | Yes |
| 11 | 51704 | GPRC5B | 1043 | -0.438 | -0.2164 | Yes |
| 12 | 54676 | GTPBP2 | 1050 | -0.447 | -0.1658 | Yes |
| 13 | 2012 | EMP1 | 1119 | -0.494 | -0.1540 | Yes |
| 14 | 604 | BCL6 | 1136 | -0.504 | -0.1036 | Yes |
| 15 | 9411 | ARHGAP29 | 1174 | -0.539 | -0.0640 | Yes |
| 16 | 26137 | ZBTB20 | 1244 | -0.622 | -0.0372 | Yes |
| 17 | 837 | CASP4 | 1245 | -0.623 | 0.0392 | Yes |
| 18 | 5106 | PCK2 | 1252 | -0.640 | 0.1134 | Yes |
Table: GSEA details [plain text format]

  

Fig 2: GSE24142\_ADULT\_VS\_FETAL\_DN2\_THYMOCYTE\_UP      
 Blue-Pink O' Gram in the Space of the Analyzed GeneSet

  

Fig 3: GSE24142\_ADULT\_VS\_FETAL\_DN2\_THYMOCYTE\_UP: Random ES distribution      
 Gene set null distribution of ES for **GSE24142\_ADULT\_VS\_FETAL\_DN2\_THYMOCYTE\_UP**
